# Supplementary material for: A positive charge region of Salmonella FliI is required for ATPase formation and efficient flagellar protein export
Source: Commun Biol. 2021 Apr 12;4:464. doi: 10.1038/s42003-021-01980-y (PMC8041783; doi:10.1038/s42003-021-01980-y)
Supplement: Supplementary file 2 — Supplementary Information [file 42003_2021_1980_MOESM2_ESM.pdf]

Supporting Information

A positive charge region of *Salmonella* FliI is required for ATPase formation and efficient flagellar protein export

Miki Kinoshita, Keiichi Namba and Tohru Minamino

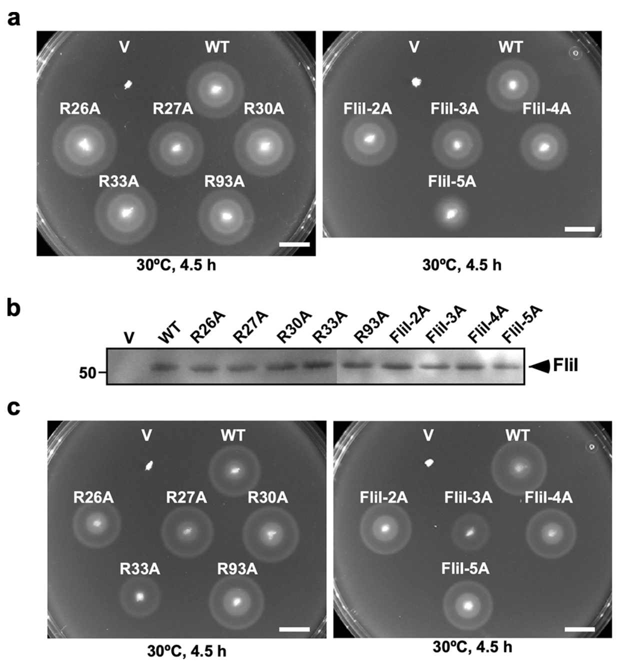

Supplementary Fig. 1. Effect of FliI mutations on motility. **(a)** Motility of MKM30 (Δ*fliI*) cells transformed with pET19b (indicated, V), pMM1701 (pET19b/His-FliI, indicated as WT), pMM1701(R26A) [pET19b/His-FliI(R26A), indicated R26A], pMM1701(R27A) [pET19b/His-FliI(R27A), indicated as R27A], pMM1701(R30A) [pET19b/His-FliI(R30A), indicated as R30A], pMM1701(R33A) [pET19b/His-FliI(R33A), indicated as R33A], pMM1701(R93A) [pET19b/His-FliI(R93A), indicated as R93A], pMM1701-2A [pET19b/His-FliI(R26A/R27A), indicated as FliI-2A], pMM1701-3A [pET19b/His-FliI(R26A/R27A/R33A), indicated as FliI-3A], pMM1701-4A [pET19b/His-FliI(R26A/R27A/R33A/R76A), indicated as FliI-4A] or pMM1701-5A [pET19b/His-FliI(R26A/R27A/R33A/R76A/R93A), indicated as Low copy, FliI-5A] in 0.35% soft agar plates containing 100 μg ml^{-1} ampicillin. Plates were incubated at 30ºC for 4.5 hours. Scale bar, 1.0 cm. **(b)** Immunoblotting, using polyclonal anti-FliI antibody, of whole cell proteins prepared from the above transformants. **(c)** Multicopy effect of FliI mutant proteins on motility of the *fliI* null mutant. Fresh MKM30 cells carrying pTrc99A (indicated as V), pMM1702 (pTrc99A/His-FliI, indicated as WT), pMM1702(R26A) [pTrc99A/His-FliI(R26A), indicated as R26A], pMM1702(R27A) [pTrc99A/His-FliI(R27A), indicated as R27A], pMM1702(R30A) [pTrc99A/His-FliI(R30A), indicated as R30A], pMM1702(R33A) [pTrc99A/His-FliI(R33A), indicated as R33A], pMM1702(R93A) [pTrc99A/His-FliI(R93A), indicated as R93A], pMM1702-2A [pTrc99A/His-FliI(R26A/R27A), indicated as FliI-2A], pMM1702-3A [pTrc99A/His-FliI(R26A/R27A/R33A), indicated as FliI-3A], pMM1702-4A [pTrc99A/His-FliI(R26A/R27A/R33A/R76A), indicated as FliI-4A], or pMM1702-5A [pTrc99A/His-FliI(R26A/R27A/R33A/R76A/R93A), indicated as FliI-5A] were inoculated onto soft agar plates containing ampicillin, and the plates were incubated at 30ºC for 4.5 hours. Scale bar, 1.0 cm.

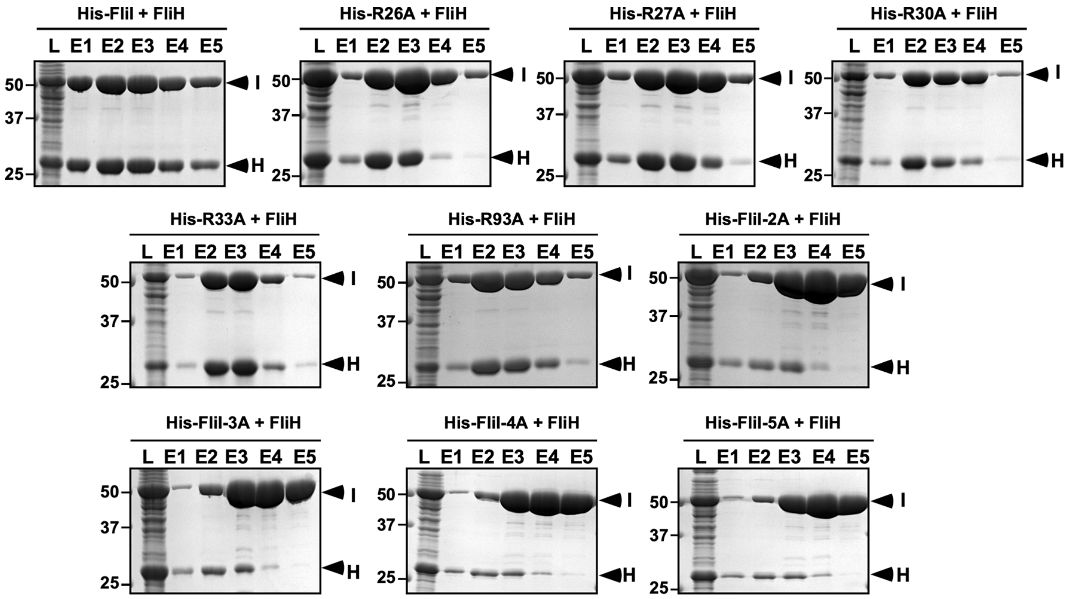

Supplementary Fig. 2. **Interaction between FliH and FliI.** Cell lysates (L) from *Salmonella* SJW1368 (Δ*cheW-flhD*) cells co-expressing FliH with either His-FliI, His-FliI(R26A), His-FliI(R27A), His-FliI(R30A), His-FliI(R33A), His-FliI(R93A), His-FliI-2A, His-FliI-3A, His-FliI-4A or His-FliI-5A were loaded onto a Ni-NTA agarose column. After extensive washing with a binding buffer (20 mM Tris-HCl, pH 8.0, 500 mM NaCl) containing 50 mM imidazole, proteins were eluted with 1 ml of the binding buffer containing 100 mM imidazole (E1), followed by 1 ml of the binding buffer containing 250 mM imidazole (E2) and finally 3 ml of the binding buffer containing 500 mM imidazole (E3–E5). The eluted fractions were analyzed by CBB staining. H and I indicate FliH and His-FliI, respectively.

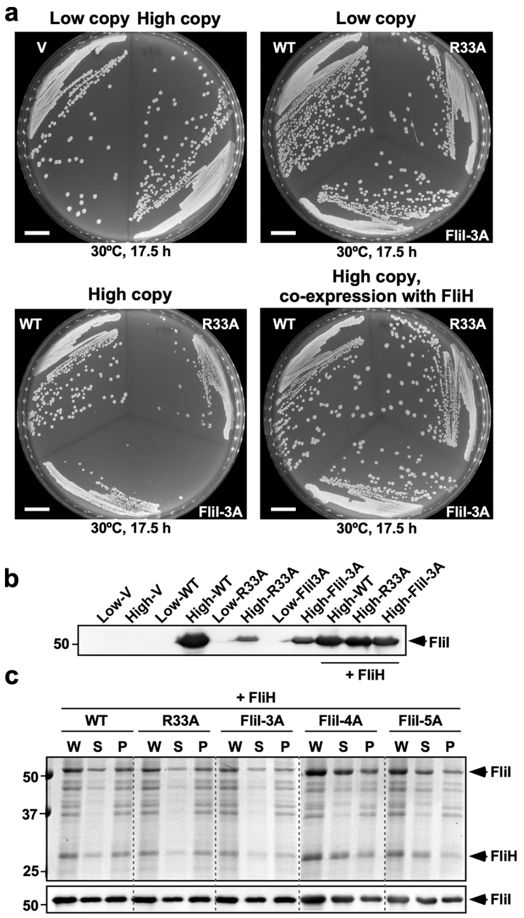

Supplementary Fig. 3. **Multicopy effect of FliI(R33A) and FliI-3A on the growth of the *fliI* null mutant.** **(a)** Cell growth measurements. Fresh colonies of *Salmonella* MKM30 (Δ*fliI*) cells carrying pET19b (indicated as Low copy, V), pTrc99A (indicated as High copy, V), pMM1701 (pET19b/His-FliI, indicated as Low copy, WT), pMM1701(R33A) [pET19b/His-FliI(R33A), indicated as Low copy, R33A], pMM1701-3A [pET19b/His-FliI(R26A/R27A/R33A), indicated as Low copy, FliI-3A], pMM1702 (pTrc99A/His-FliI, indicated as High copy, WT), pMM1702(R33A) [indicated as pTrc99A/His-FliI(R33A), High copy, R33A], pMM1702-3A [pTrc99A/His-FliI(R26A/R27A/R33A), indicated as High copy, FliI-3A], pMKM1702iH (pTrc99A/His-FliI + FliH, indicated as High copy, WT), pMKM1702(R33A)iH (pTrc99A/His-FliI(R33A) + FliH, indicated as High copy, R33A) or pMKM1702-3AiH [pTrc99A/His-FliI(R26A/R27A/R33A) + FliH, indicated as High copy, FliI-3A] were inoculated onto L-broth agar plate containing ampicillin and incubated at 30ºC for 17.5 hours. Scale bar, 1.0 cm. **(b)** Immunoblotting, using polyclonal anti-FliI antibody, of whole cell proteins prepared from the same transformants. **(c)** Effect of co-expression of FliH on the solubility of FliI. Coomassie blue-stained gels (upper panel) and immunoblots (lower panel), using polyclonal FliI antibody, of whole cellular (W), soluble (S) and insoluble (P) fractions prepared from MKM30 cells carrying pMKM1702iH (pTrc99A/His-FliI + FliH, indicated as WT), pMKM1702(R33A)iH (pTrc99A/His-FliI(R33A) + FliH, R33A), pMKM1702-3AiH [pTrc99A/His-FliI(R26A/R27A/R33A) + FliH, indicated as FliI-3A], pMKM1702-4AiH [pTrc99A/His-FliI(R26A/R27A/R33A/R76A) + FliH, indicated as FliI-

4A] or pMKM1702-5AiH [pTrc99A/His-FliI(R26A/R27A/R33A/R76A/R93A) + FliH, indicated as FliI-5A].

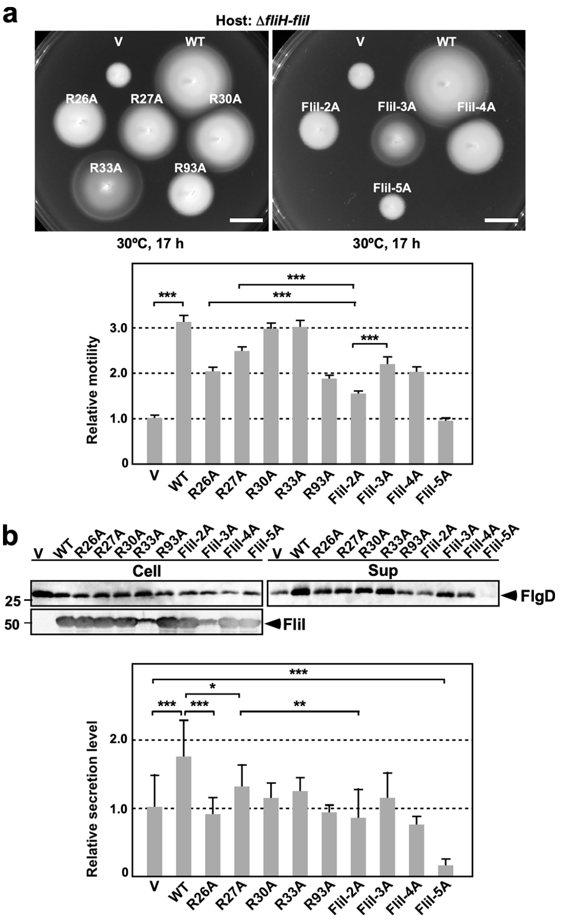

Supplementary Fig. 4. Characterization of FliI mutant variants in the absence of FliH. (a) Motility of MMHI001 (Δ*fliH-fliI*) cells transformed with pTrc99A (V), pMM1702 (WT), pMM1702(R26A) (R26A), pMM1702(R27A) (R27A), pMM1702(R30A) (R30A), pMM1702(R33A) (R33A), pMM1702(R93A) (R93A), pMM1702-2A (FliI-2A), pMM1702-3A (FliI-3A), pMM1702-4A (FliI-4A) or pMM1702-5A (FliI-5A) in 0.35% soft agar plates containing 100 μg ml^{-1} ampicillin. Plates were incubated at 30ºC for 17 hours. The diameter of the motility ring of 5 colonies of each transformant was measured. The average diameter of the motility ring of the vector control was set to 1.0, and then relative diameter of the motility ring of each transformants was calculated. Vertical bars indicate standard deviations. Scale bar, 1.0 cm. **(b)** Immunoblotting, using polyclonal anti-FlgD (1st row) or anti-FliI (2nd row) antibody, of whole cell proteins and culture supernatant fractions prepared from the above transformants. To clearly see the FlgD secretion by the vector control strain, each transformants were grown overnight at 30ºC in L-broth. Relative secretion levels of FlgD were measured. These data are average of seven independent experiments. Vertical bars indicate standard deviations. Comparisons between datasets were performed using a two-tailed Student's *t*-test. A *P* value of < 0.05 was considered to be statistically significant difference. *, $P < 0.05$; **, $P < 0.01$; ***, $P < 0.001$.

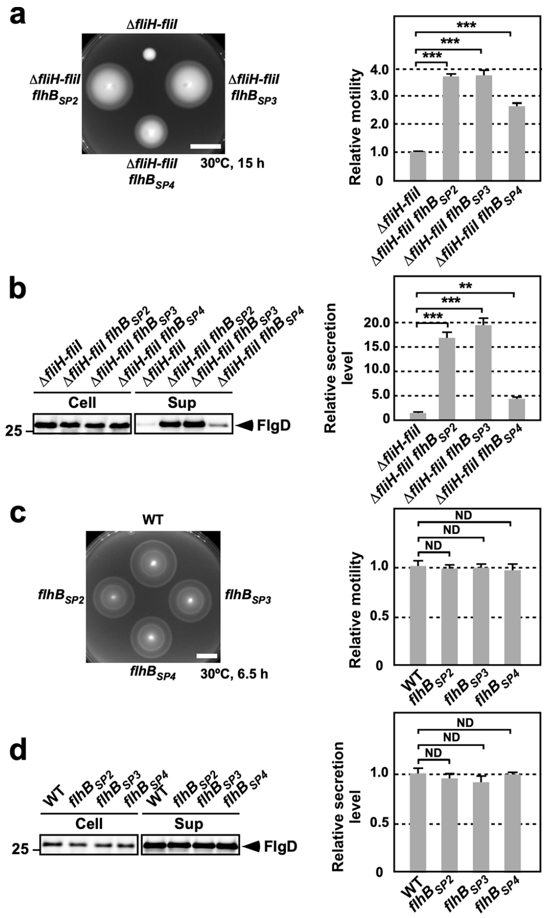

Supplementary Fig. 5. Characterization of gain-of-function mutations in FlhB. (a) Motility of the *Salmonella* MMHI001 (Δ*fliH-fliI*), MMHI001-5A-SP2 (Δ*fliH-fliI flhB$_{SP2}$*) MMHI001-5A-SP3 (Δ*fliH-fliI flhB$_{SP3}$*) and MMHI001-5A-SP4 (Δ*fliH-fliI flhB$_{SP4}$*) strains in 0.35% soft agar. Plates were incubated at 30ºC for 15 hours. The diameter of the motility ring of 5 colonies of each strain was measured. Scale bar, 1.0 cm. The average diameter of the motility ring of the Δ*fliH-fliI* strain was set to 1.0, and then relative diameter of the motility ring of each suppressor mutant strain was calculated. Vertical bars indicate standard deviations. Scale bar, 1.0 cm. **(b)** Immunoblotting, using polyclonal anti-FlgD antibody, of whole cell proteins and culture supernatant fractions prepared from the above strains. Relative secretion levels of FlgD were measured. These data are average of three independent experiments. Vertical bars indicate standard deviations. **(c)** Motility of the SJW1103 (WT), MMB5A-SP2 (*flhB$_{SP2}$*) MMB5A-SP3 (*flhB$_{SP3}$*) and MMB5A-SP4 (*flhB$_{SP4}$*) stains in 0.35% soft agar at 30ºC for 6.5 hours. The diameter of the motility ring of 5 colonies of each strain was measured. Scale bar, 0.5 cm. The average diameter of the motility ring of the wild-type strain was set to 1.0, and then relative diameter of the motility ring of each *flhB* mutant strain was calculated. Vertical bars indicate standard deviations. **(d)** Immunoblotting, using polyclonal anti-FlgD antibody, of whole cell proteins and culture supernatant fractions prepared from the same strains. Relative secretion levels of FlgD were measured. These data are average of three independent experiments. Vertical bars indicate standard deviations. Comparisons between datasets were performed using a two-tailed Student's *t*-test. A

P value of < 0.05 was considered to be statistically significant difference. **, *P* < 0.01; ***, *P* < 0.001; ND, no statistical difference.

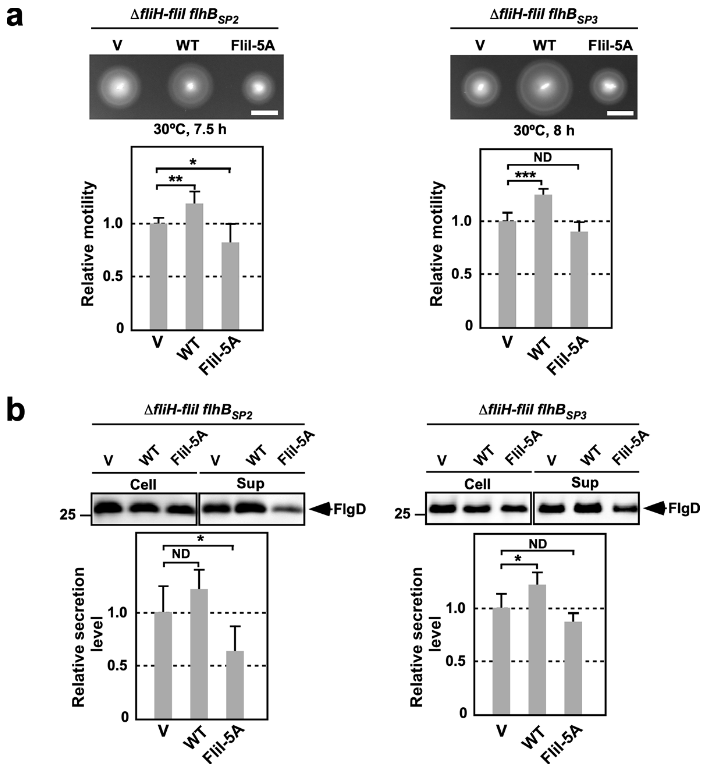

Supplementary Fig. 6. Multicopy effect of FliI-5A on motility of and flagellar protein export by the Δ*fliH-fliI flhB*$_{SP3}$ and Δ*fliH-fliI flhB*$_{SP4}$ cells. Motility of the Δ*fliH–fliI flhB*$_{sp2}$ (left panels) and Δ*fliH–fliI flhB*$_{sp3}$ (right panels) cells carrying with pTrc99A (V), pMM1702 (WT) or pMM1702-5A (FliI-5A) in soft agar. Plates were incubated at 30ºC. The diameter of the motility ring of 5 colonies of each strain was measured. The average diameter of the motility ring of the vector control was set to 1.0, and then relative diameter of the motility ring of each transformants was calculated. Vertical bars indicate standard deviations. Scale bar, 0.5 cm. **(b)** Immunoblotting, using polyclonal anti-FlgD antibody, of whole cell proteins and culture supernatant fractions prepared from the above strains. Relative secretion levels of FlgD were measured. These data are average of three independent experiments. Vertical bars indicate standard deviations. Comparisons between datasets were performed using a two-tailed Student's *t*-test. A *P* value of < 0.05 was considered to be statistically significant difference. *, $P < 0.05$; **, $P < 0.01$; ***, $P < 0.001$; ND, no statistical difference.

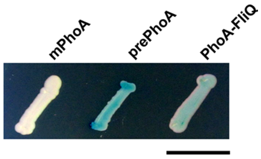

Supplementary Fig. 7. Effect of a fusion of FliQ to the C-terminus of PhoA on the PhoA phosphatase activity. *Salmonella* TH12991 (Δ*phoN*) cells were transformed with pMKM10001 (pTrc99A/mPhoA), pMKM10002 (pTrc99A/prePhoA) or pMKM10003 (pTrc99A/PhoA–FliQ), and then fresh transformants were inoculated onto BCIP indicator plates and incubated at 30°C for 18 hours. Blue colonies indicate that PhoA is located within the periplasm whereas white colonies show that PhoA is in the cytoplasm. Scale bar, 1.0 cm.

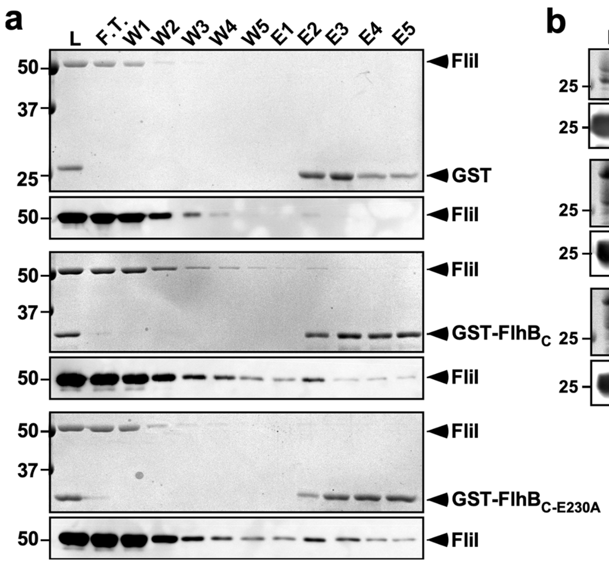

Supplementary Fig. 8. Effect of the *flhB(E230A)* mutation on interactions of FlhB$_C$ with FliI and FlgD (a) Interaction between FlhB$_C$ and FliI. Purified His-FliI was mixed with GST, GST-FlhB$_C$, or GST-FlhB$_{C\text{-}E230A}$ and dialyzed overnight against PBS. These mixtures (L) were subjected to GST affinity chromatography. Flow through fraction (F.T.), wash fractions (W), and elution fractions (E) were analyzed by both CBB staining (upper panel) and immunoblotting with polyclonal anti-FliI antibody (lower panel). The positions of molecular mass markers are shown on the left. **(b)** Interaction between FlhB$_C$ and FlgD. Cell lysates (indicated as L) prepared from *Salmonella* SJW1368 (Δ*cheW-flhD*) cells expressing GST, GST-FlhB$_C$ or GST-FlhB$_{C\text{-}E230A}$ were mixed with those from *E. coli* BL21 (DE3) cells producing FlgD, and each mixture was loaded onto a GST column. After washing with PBS, proteins were eluted with 50 mM Tris-HCl, pH 8.0, 10 mM reduced glutathione. Elution fractions were analyzed by both CBB staining (upper panel) and immunoblotting with polyclonal anti-FlgD antibody (lower panel).

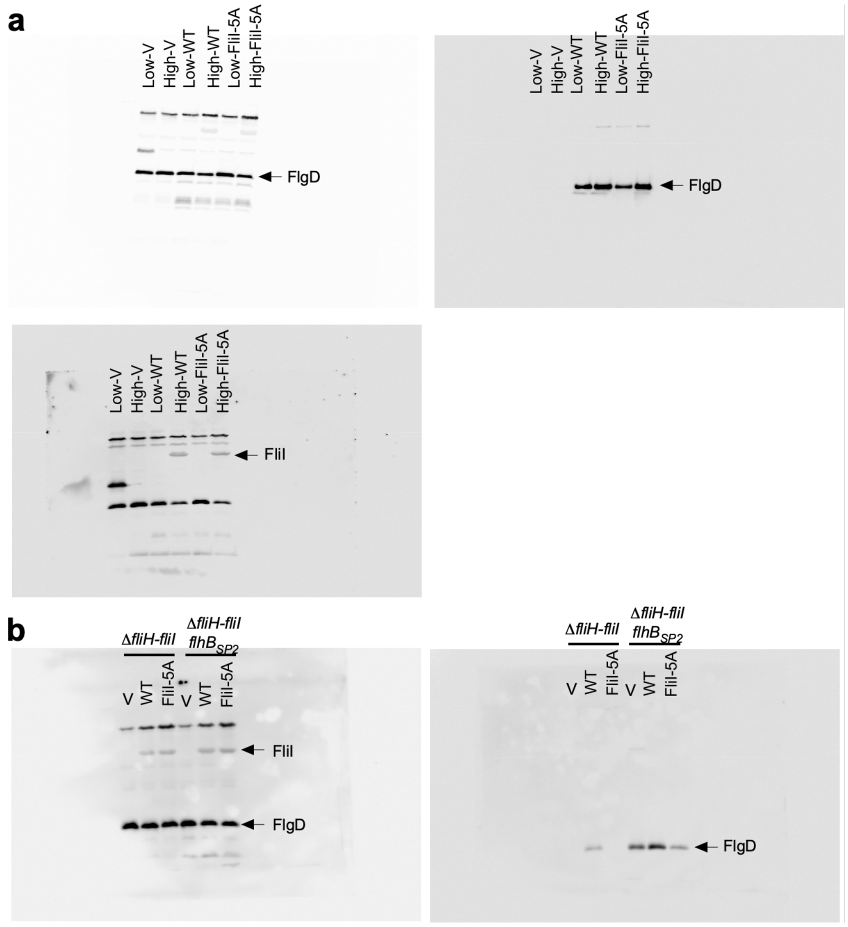

Supplementary Fig. 9. Original immunoblots shown in Figures 3b and 3d.

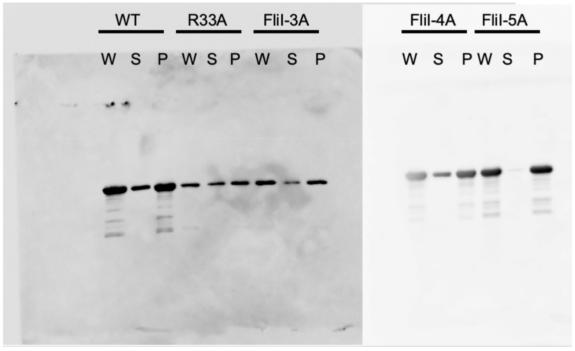

Supplementary Fig. 10. Original immunoblots shown in Figure 4b.

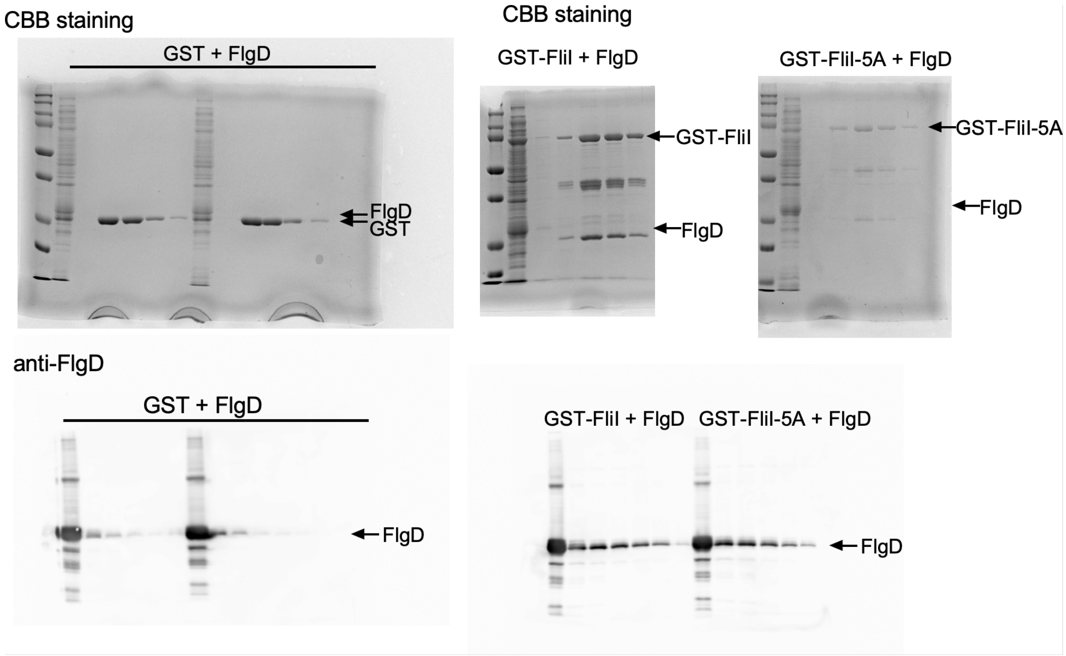

Supplementary Fig. 11. Original CBB-stained gels and immunoblots shown in Figure 6.

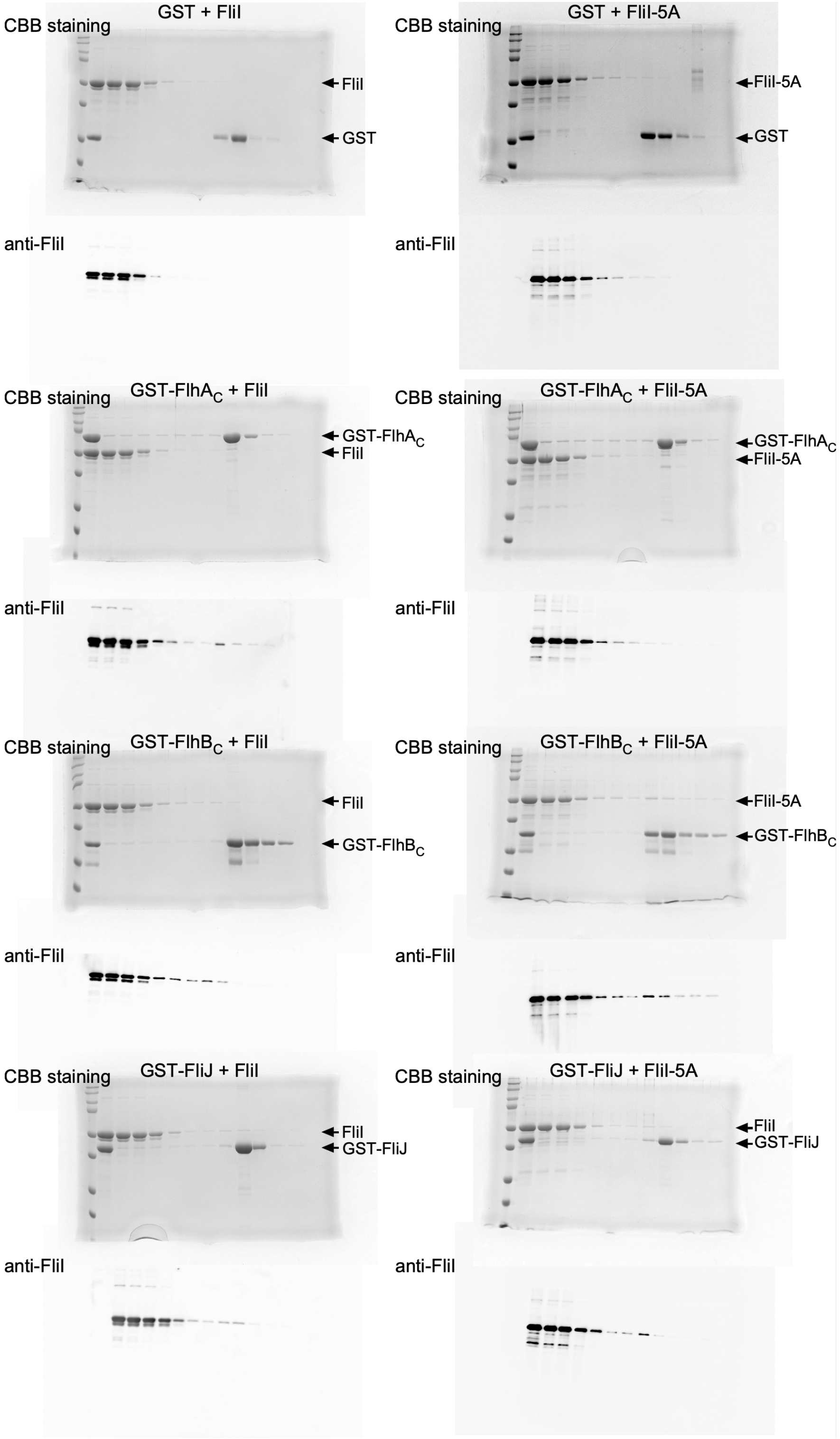

Supplementary Fig. 12. Original CBB-stained gels and immunoblots shown in Figure 7.

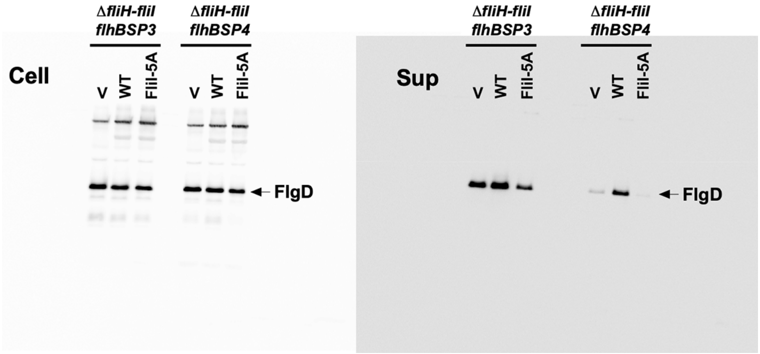

Supplementary Fig. 13. Original immunoblots shown in Figure 8d.

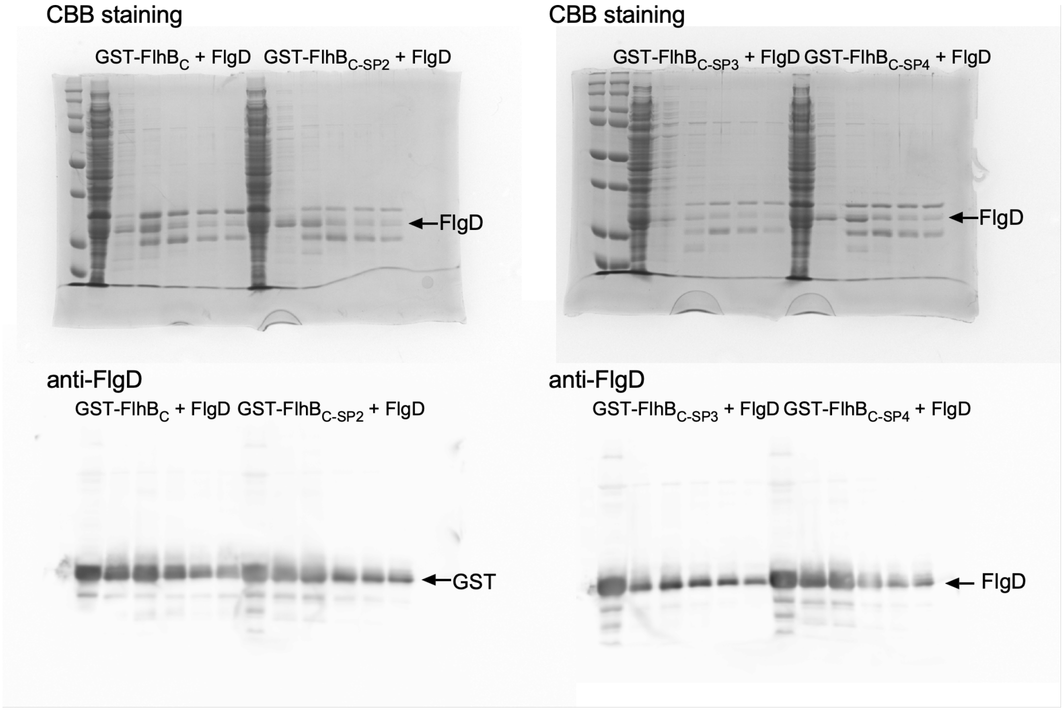

Supplementary Fig. 14. Original CBB-stained gels and immunoblots shown in Figure 8e.

Supplementary Table 1. Strains and plasmids used in this study

Strain/Plasmid	Relevant characteristics	References
E. coli		
BL21(DE3) Star	Overexpression of proteins	Novagen
Salmonella		
SJW1103	Wild-type for motility and chemotaxis	1
SJW1368	Δ(*cheW-flhD*); master operon mutant	2
MKM30	Δ*fliI*	3
MMHI001	Δ*fliH–fliI*	4
MMHI001-5A-SP2	Δ*fliH-fliI* $flhB_{SP2}$	This study
MMHI001-5A-SP3	Δ*fliH-fliI* $flhB_{SP3}$	This study
MMHI001-5A-SP4	Δ*fliH-fliI* $flhB_{SP4}$	This study
MMB5A-SP2	$flhB_{SP2}$	This study
MMB5A-SP3	$flhB_{SP3}$	This study
MMB5A-SP4	$flhB_{SP4}$	This study
TH12991	Δ*phoA*	Kelly T. Hughes
Plasmids		
pET19b	Expression vector	Novagen
pGEX-6p-1	Expression vector	GE Healthcare
pTrc99A	Expression vector	GE Healthcare
pGKK1702	pGEX-6p-1/ GST-FliI	5
pGKK1702-5A	pGEX-6p-1/ GST-FliI(R26A/R27A/R33A/R76A/R93A)	This study
pMM1701	pET19b/ His-FliI	6
pMM1702	pTrc99A/ His-FliI	6
pMMHA1001	pGEX-6p-1/ GST-$FlhA_C$	7
pMMHB1001	pGEX-6p-1/ GST-$FlhB_C$	7
pMMHB1001-SP2	pGEX-6p-1/ GST-$FlhB_{C\text{-}SP2}$	This study
pMMHB1001-SP3	pGEX-6p-1/ GST-$FlhB_{C\text{-}SP3}$	This study
pMMHB1001-SP4	pGEX-6p-1/ GST-$FlhB_{C\text{-}SP4}$	This study
pMMHB1001(E230A)	pGEX-6p-1/ GST-$FlhB_{C\text{-}E230A}$	This study
pMMJ1001	pGEX-6p-1/ GST-FliJ	8
pMM1701(R26A)	pET19b/ His-FliI(R26A)	This study
pMM1702(R26A)	pTrc99A/ His-FliI(R26A)	This study
pMM1701(R27A)	pET19b/ His-FliI(R27A)	This study
pMM1702(R27A)	pTrc99A/ His-FliI(R27A)	This study
pMM1701(R30A)	pET19b/ His-FliI(R30A)	This study
pMM1702(R30A)	pTrc99A/ His-FliI(R30A)	This study
pMM1701(R33A)	pET19b/ His-FliI(R33A)	This study
pMM1702(R33A)	pTrc99A/ His-FliI(R33A)	This study
pMM1701(R93A)	pET19b/ His-FliI(R93A)	This study
pMM1702(R93A)	pTrc99A/ His-FliI(R93A)	This study
pMM1701-2A	pET19b/ His-FliI(R26A/R27A)	This study
pMM1702-2A	pTrc99A/ His-FliI(R26A/R27A)	This study
pMM1701-3A	pET19b/ His-FliI(R26A/R27A/R33A)	This study
pMM1702-3A	pTrc99A/ His-FliI(R26A/R27A/R33A)	This study
pMM1701-4A	pET19b/ His-FliI(R26A/R27A/R33A/R76A)	This study
pMM1702-4A	pTrc99A/ His-FliI(R26A/R27A/R33A/R76A)	This study
pMM1701-5A	pET19b/ His-FliI(R26A/R27A/R33A/R76A/R93A)	This study
pMM1702-5A	pTrc99A/ His-FliI(R26A/R27A/R33A/R76A/R93A)	This study
pMKM1702iH	pTrc99A/ His-FliI + FliH	9
pMKM1702(R26A)iH	pTrc99A/ His-FliI(R26A) + FliH	This study
pMKM1702(R27A)iH	pTrc99A/ His-FliI(R27A) + FliH	This study
pMKM1702(R30A)iH	pTrc99A/ His-FliI(R30A) + FliH	This study
pMKM1702(R33A)iH	pTrc99A/ His-FliI(R33A) + FliH	This study
pMKM1702(R93A)iH	pTrc99A/ His-FliI(R93A) + FliH	This study

pMKM1702-2AiH	pTrc99A/ His-FliI(R26A/R27A) + FliH	This study
pMKM1702-3AiH	pTrc99A/ His-FliI(R26A/R27A/R33A) + FliH	This study
pMKM1702-4AiH	pTrc99A/ His-FliI(R26A/R27A/R33A/R76A) + FliH	This study
pMKM1702-5AiH	pTrc99A/ His-FliI(R26A/R27A/R33A/R76A/R93A) + FliH	This study
pMKM10001	pTrc99A/ mPhoA	This study
pMKM10002	pTrc99A/ prePhoA	This study
pMKM10003	pTrc99A/ PhoA–FliQ	This study
pMKM10004	pTrc99A /PhoA–FlhB	This study
pMKM10005	pTrc99A/ $FlhB_{(1-59)}$–PhoA	This study
pMKM10006	pTrc99A/ $FlhB_{(1-132)}$–PhoA	This study
pMKM10007	pTrc99A/ $FlhB_{(1-184)}$–PhoA	This study
pMKM10008	pTrc99A/ $FlhB_{(1-212)}$–PhoA	This study
pMKM10009	pTrc99A/ PhoA–FlhA	This study
pMKM10010	pTrc99A/ $FlhA_{(1-44)}$–PhoA	This study
pMKM10011	pTrc99A/ $FlhA_{(1-65)}$–PhoA	This study
pMKM10012	pTrc99A/ $FlhA_{(1-93)}$–PhoA	This study
pMKM10013	pTrc99A/ $FlhA_{(1-196)}$–PhoA	This study
pMKM10014	pTrc99A/ $FlhA_{(1-236)}$–PhoA	This study
pMKM10015	pTrc99A/ $FlhA_{(1-278)}$–PhoA	This study
pMKM10016	pTrc99A/ $FlhA_{(1-306)}$–PhoA	This study
pMKM10017	pTrc99A/ $FlhA_{(1-339)}$–PhoA	This study

Supplementary References

1. Yamaguchi, S., Fujita, H., Sugata, K., Taira, T. & Iino, T. Genetic analysis of *H2*, the structural gene for phase-2 flagellin in *Salmonella*. *J. Gen. Microbiol.* **130**, 255–265 (1984).
2. Ohnishi, K., Ohto, Y., Aizawa, S.-I., Macnab, R.M. & Iino, T. FlgD is a scaffolding protein needed for flagellar hook assembly in *Salmonella typhimurium. J. Bacteriol.* **176**, 2272–2281 (1994).
3. Minamino, T., González-Pedrajo, B., Kihara, M., Namba, K. & Macnab, R. M. The ATPase FliI can interact with the type III flagellar protein export apparatus in the absence of its regulator FliH. *J. Bacteriol.* **185**, 3983–3988 (2003).
4. Minamino, T. *et al*. Oligomerization of the bacterial flagellar ATPase FliI is controlled by its extreme N-terminal region. *J. Mol. Biol.* **360**, 510–519 (2006).
5. Kazetani, K., Minamino, T., Miyata, T., Kato, T. & Namba, K. ATP-induced FliI hexamerization facilitates bacterial flagellar protein export. *Biochem. Biophys. Res. Commun.* **388**, 323–327 (2009).
6. Minamino, T. & Macnab, R. M. Interactions among components of the *Salmonella* flagellar export apparatus and its substrates. *Mol. Microbiol.* **35**, 1052–1064 (2000).
7. Minamino, T. *et al*. Role of the C-terminal cytoplasmic domain of FlhA in bacterial flagellar type III protein export. *J. Bacteriol.* **192**, 1929–1936 (2010).
8. Minamino, T. *et al*. Roles of the extreme N-terminal region of FliH for efficient localization of the FliH-FliI complex to the bacterial flagellar type III export apparatus. *Mol. Microbiol.* **74**, 1471–1483 (2009).
9. Imada, K., Minamino, T., Uchida, Y., Kinoshita, M. & Namba, K. Insight into the flagella type III export revealed by the complex structure of the type III ATPase and its regulator. *Proc. Natl. Acad. Sci. USA* **113**, 3633–3638 (2016).
